# Thymoglobulin – new approaches to optimal outcomes

**Published:** 2009

**Authors:** Andreea Delia Moiceanu, Anca Maria Popp, Ioanel Sinescu

**Affiliations:** * Fundeni Clinical Institute, Second Department of Hematology, Bucharest, Romania; ** Fundeni Clinical Institute, Third Department of Hematology, Bucharest, Romania; *** Fundeni Clinical Institute, Center for Urology, Dialysis and Kidney Transplantation

## Abstract

Thymoglobulin has a proven safety and efficacy profile both as treatment of acute rejection and as induction therapy in organ transplantation. The most common adverse events associated with Thymoglobulin are cytokine release syndrome, thrombocytopenia, and lymphopenia. Results of early studies showed an increased rate of cytomegalovirus disease associated with Thymoglobulin treatment, but recent studies indicate that routine administration of modern antiviral prophylaxis can reduce this risk. More research comparing Thymoglobulin with basiliximab will help individualize regimens by matching the choice of induction agent with the risk profile of each transplant recipient. The proven efficacy and safety profile of Thymoglobulin provides an excellent starting point for future investigations.[**50**]

Horse ATG (hATG) or Thymoglobulin + Cyclosporine are an efficacious treatment for aplastic anemia. Due to its higher potency Thymoglobulin may be superior to hATG, but further studies are required for confirmation.[**38**]

GvHD prophylaxis with Thymoglobulin may result in less acute and chronic GvHD, lower TRM, improved survival and quality of life in myeloablative or reduced intensity conditioning protocols in patients receiving hematopoietic stem cells from related or unrelated donors.

Attributable to its polyclonal nature, Thymoglobulin provides multifaceted immunomodulation suggesting that its use should be included in the immunosuppressant therapeutic armamentarium to help reduce the incidence of organ rejection and GvHD,[**5**] and for treatment of aplastic anemia.

## Introduction

Immunosuppressive properties of polyclonal antithymocyte globulins (ATG) were first described in the 1950s,[**1**] and ATG have been widely used for more than 30 years.[**2**] Recent findings demonstrate that ATG can provide a wide spectrum of immunomodulation, suggesting that their use in immunosuppression may help in reducing the incidence of organ rejection, improving patients’ outcome after hematopoietic stem cell transplantation,[**3**] and treating autoimmune mediated disease, i.e. aplastic anemia. 

ATG is a mixture of different antibody specificities, which induces an extremely effective dose-dependent T-cell depletion in blood and lymphoid tissues via complement-dependent cytotoxicity, antibody dependent cellular cytotoxicity, and apoptosis.[**4**] Currently there are three different ATGs commercially available: Human thymocytes are used as the immunogenic to produce Atgam® (Pharmacia & Upjohn, NY, USA) in horses and Thymoglobulin® (Genzyme Polyclonals, S.A.S. Marcy L’Etoile, France) in rabbits, respectively; a Jurkat cell line is used to produce ATG-Fresenius® (Fresenius Biotech GmbH, Graefelfing, Germany) in rabbits.[**4**] Despite sharing some common properties, the commercially available ATG products are strictly different drugs.[**5**] Immunosuppressive activity varies significantly from one preparation to the other, resulting in quite different dosages. Among these products, Thymoglobulin is probably the most potent, and the most extensively studied ATG.[**5**,**6**] This review describes the clinical use of Thymoglobulin in organ transplantation and hematology/oncology.

## Mechanisms of action

The role of Thymoglobulin in the prevention and treatment of allograft rejection, graft versus- host disease (GVHD), and treatment of aplastic anemia (AA) is well established. Recent investigations have shown that Thymoglobulin does not only deplete T-cells, but modulates various lymphocyte surface antigens and interferes with the function of a number of different immune effector cells, including B cells, dendritic cells, natural killer (NK) T cells, and regulatory T cells (Tregs).[**7**]

## Solid organ transplantation: Prevention of rejection (induction)

The risk of organ rejection is bigger immediately (weeks to months) after transplantation. It declines during the first year and further on, but it is present through the whole life of the graft.[**8**] Thymoglobulin is indicated for prevention of graft rejection in organ transplantation (induction); dosage 1 to 1,5 mg/kg/day for 2 to 9 days (2 to 5 days in heart transplantation).[**9**] In the US, antibody induction is used in the majority (>70%) of kidney and almost 50% of thoracic organ transplantations, and Thymoglobulin is the most frequently used induction agent.[**10**] It has the following roles in organ transplant recipients: reduction of the incidence of acute rejection, prevention of ischemia reperfusion injury and delayed graft function, and minimization of calcineurin inhibitors (CNIs) and/or corticosteroids.[**13**,**18**,**23**-**25**] 

## Thymoglobulin induction versus no induction

In two randomized, prospective trials Thymoglobulin was shown to decrease the rate of acute rejection in kidney transplant patients compared to no induction (15,1% vs. 25,4%; 15,2% vs. 30,4% respectively, p<0.001 in both studies). In these early studies, the incidences of leucopenia, thrombocytopenia, fever, and cytomegalovirus infection were significantly higher in the Thymoglobulin groups.[**11**,**12**] A retrospective analysis in living donor kidney transplantation (n=214) in a single center versus a national cohort showed a significant benefit of Thymoglobulin induction vs. no antibody induction in a low risk patient population. Five years patient survival was 96% vs. 90% (p=0,03), and acute rejection at one year was 2% vs. 21% (p<0,001). Thymoglobulin was well tolerated with very few infections, and a low incidence of malignancy.[**13**]

## Thymoglobulin induction versus other ATG induction

In a prospective, double blind trial event free survival (defined as freedom from death, graft loss, or biopsy proven acute rejection - BPAR) after one (94% vs. 63% p=0,0005), five (73% vs. 33% p<0,001), and ten (48% vs. 29% p=0,011) years was significantly higher in Thymoglobulin treated patients (n=48) receiving a kidney transplant compared to Atgam (n=24). There were no post transplant lymphoproliferative disorder (PTLD) in the Thymoglobulin group and two cases in the Atgam group.[**14**] 

One prospective, randomized trial compared induction with Thymoglobulin (n=28) and ATG Fresenius (n=30) in kidney transplant recipients. Acute rejection after one year was numerically lower in the Thymoglobulin group (14,2% vs. 26,6%; ns). Thymoglobulin patients experienced a lower incidence of infections, lower white blood cell (WBC) counts while maintaining hemoglobin levels better.[**15**] In a single-center, retrospective study Thymoglobulin induction (n=65) seemed to be connected with higher rates of CMV disease, malignancy, and death than ATG Fresenius (n=129).[**16**] However limitations of this study are the variable doses of Thymoglobulin (17 – 6 mg/kg total),[**4**] and lack of antiviral prophylaxis for the longest period of time.

A retrospective analysis compared the long-term benefits of induction with Thymoglobulin (n=342) and ATG Fresenius (n=142) in heart transplantation from 1984 to 1996. Five year patient survival rate was significantly higher in the Thymoglobulin group (76% versus 60%; p<0,01); 72% versus 42% (p<0,01) of patients were free from acute rejection, less and less severe recurrent rejections were observed. Viral infections (53% vs. 39%; p<0,05), but not cytomegalovirus (CMV) infections (17% vs. 13%), were more frequently observed in the Thymoglobulin group. Post transplant lymphoproliferative disorders (PTLD) were comparable. The authors concluded, that the two rabbit ATGs have different immunosuppressive potency, and that Thymoglobulin is currently the most powerful induction agent in heart transplantation.[**17**]

## Thymoglobulin versus anti-IL-2R antibodies

In a prospective, randomized trial comparing induction with Thymoglobulin (n=141) and basiliximab (Simulect®, Novartis Pharmaceuticals, East Hanover, NJ, USA ; n=137) in patients receiving a kidney transplant from a marginal donor, BPAR was lower in the Thymoglobulin group after one (15,6% vs. 25,5% p=0,02)[**18**] and five years in the US cohort (15% vs. 25%).[**19**] Moreover acute rejection following Thymoglobulin induction was less severe: Rejection rates requiring antibody treatment were 1,4% vs. 8% (p=0,005) at one year[**18**] and 3% vs. 12% in the US cohort after five years (p=0,05).[**19**] The authors concluded, that Thymoglobulin and basiliximab have equivalent but different safety profiles, and require appropriate antibacterial and antiviral prophylaxis strategies.[**18**] CMV disease occurred more frequently in patients treated with basiliximab (17,5% vs. 7,8%; p=0,02; 17% vs. 7% US cohort after five years, respectively; p = 0.02),[**18**,**19**] whereas the rate of infections was higher in the Thymoglobulin group (85.8% vs. 75.2%, P = 0.03).[**18**] However an economic analysis showed, that in Thymoglobulin patients 12 months post-transplant, treatment costs were continuously lower. The combination of lower costs and improved outcomes make Thymoglobulin both clinically and economically preferred over basiliximab in patients receiving a kidney from a marginal donor.[**20**]

In another prospective randomized trial comparing Thymoglobulin (n=113) and daclizumab (Zenapax®, Roche AG, Basel, Switzerland; n=114) in high risk renal transplant patients with triple maintenance therapy, BPAR after one year was significantly lower in the Thymoglobulin group (19,5% vs. 29,8%; p=0,043).[**21**] Both studies showed no statistical difference between Thymoglobulin and anti-IL-2R antibodies in terms of patient- and graft survival.[**18**,**19**,**21**]

## Steroid and CNI sparing regimens

Today, death with a functioning graft and chronic allograft nephropathy (CAN) are major causes for late graft loss.[**22**] Steroids and CNI have been cornerstones for the maintenance of immunosuppression, but are associated with side effects affecting graft and patient survival and the quality of life.[**22**]Thymoglobulin induction has proven success in CNI and steroid minimization strategies. 

A prospective study (n=150) showed excellent results of Thymoglobulin induction in a CNI–free maintenance and steroid-tapering protocol. Patients were randomized to either a sirolimus- based or a cyclosporine A (CsA) -based regimen. All patients received mycophenolate mofetil (MMF) and a 6-month course of corticosteroids. At the 12-months follow-up, 88% of patients were steroid free. No significant differences were observed in patient survival (97% in each treatment group), graft survival (90% vs. 93%) or acute rejection (14.3% vs. 8.6%).[**23**]

In a retrospective study of cadaveric renal transplant recipients treated with Thymoglobulin (high immunological risk, n=30) or basiliximab (high and low risk n=115), maintenance with sirolimus and prednisone, and delayed introduction of reduced-dose CsA, BPAR at 3 months in high-immune responders was lower in patients receiving Thymoglobulin (3% vs. 26%; p=0.01). Serum creatinine was higher with basiliximab at 3, 6, and 12 months (p<0.02). Only Thymoglobulin showed an excellent result when CsA initiation was delayed for more than two weeks (0% vs. 24%).[**24**]

A single center trial of prednisone-free maintenance immunosuppression using Thymoglobulin induction and CsA/MMF or tacrolimus/sirolimus (TAC/SRL) in 589 patients showed an excellent five year patient and graft survival (91% and 84%), low incidence of acute rejection, and stable kidney function (serum creatinine 1,7±0,8 mg/dL). Steroid-related side effects like CMV infection and post transplant diabetes were minimized compared to the historic controls (p<0,0001). Thymoglobulin induction plus elimination of prednisone should be considered at least in low risk recipients.[**25**]

## Treatment of steroid resistant acute rejection

Treatment of acute rejection requires a short course of more intensive immunosuppressive therapy. First-line therapy for acute rejection is usually high dose intravenous corticosteroids. In case of steroid resistant acute rejection alternative treatments are necessary, which can be either ATG or the monoclonal antibody muromonab-CD3 (OKT3®; Ortho Biotech, Raritan NJ, USA).[**26**]

Thymoglobulin is indicated for the treatment of graft rejection in organ transplantation with a recommended dosage of 1,5 mg/kg/day for 3 to 14 days.[**9**]

A double-blind randomized trial showed superiority of over Atgam in reversal of acute rejection (88% vs. 76% p=0,027) and prevention of recurrent rejection (17% vs. 36% p=0,011) in patients who received a renal transplant. Both drugs had a similar side effect profile. The enhanced clinical efficacy of Thymoglobulin was explained by higher affinity of rabbit IgG subtype to human lymphocytes, less batch-to-batch variability, longer half-life, and more profound and longer lasting lymphocyte depletion compared to horse ATG.[**27**] Due to less frequent treatments of recurrent rejection and less frequent return to dialysis, Thymoglobulin provided significant cost savings.[**28**]

A randomized clinical trial comparing Thymoglobulin (n=31) and OKT 3 (n=29) in treatment of steroid resistant acute rejection in kidney transplant patients showed a trend in favor of Thymoglobulin (13% vs. 23% overall graft failures; 89% vs. 81% 1-year graft survival). Fever, mainly due to first dose syndrome, occurred more frequently in the OKT 3 group (52% vs. 6%, p= 0.001).[**29**] Similar efficacy of ATG or OKT 3 treatment and a fewer side effects related to first dose syndrome were reported in a meta-analysis of 21 clinical trials.[**30**]

## Thymoglobulin in hematology Treatment of AA and MDS

Aplastic anemia (AA), the paradigm of human bone marrow failures,[**31**] is a rare, potentially life-threatening failure of haemopoiesis characterized by pancytopenia and bone marrow aplasia.[**32**] Most cases of AA are acquired, although very rare inherited forms exist.[**32**] Acquired AA can occur in any age group, and in most cases results from an autoimmune attack against hematopoietic stem cells.[**31**] Immunosuppressive therapy with ATG + Cyclosporine is treatment of choice in patients above the age of 50 years, for patients who lack an HLA identical donor, and for patients with non-severe aplastic anemia.[**33**] Thymoglobulin is indicated for the treatment of aplastic anemia; recommended dosage between 2,5 and 3,5 mg/kg/day for 5 consecutive days.[**9**] For historical reasons most European studies in AA have been carried out using Lymphoglobuline® (Genzyme Polyclonals, S.A.S. Marcy L’Etoile, France), a horse ATG from the same manufacturer, which was available on the market 16 years earlier than Thymoglobulin.

The current EBMT guidelines recommend either horse or rabbit ATG in first line treatment of AA.[**34**] First line use of Thymoglobulin is recommended in the British Committee for Standards in Hematology (BCSH) guidelines for AA, because Lymphoglobuline is no longer available.[**35**] Lymphoglobuline (horse ATG) and Thymoglobulin source of antigen has been identical, both derived from human thymocytes and having comparable biological effects. Two clinical trials have shown similar response rates for both ATG in bone marrow failure.[**36**,**37**] Thymoglobulin shows excellent response (between 63,4% and 92%) and good safety profile in first line treatment of SAA[**38**] and has significant activity (33% and 42%) in low-risk MDS.[**38**,**39**] Retreatment with Thymoglobulin in patients not responding to a first course with Lymphoglobuline was associated with excellent response (77%) and survival rates (93% at 912 days follow up), and without relapse.[**40**]

The unsatisfactory result of ATG-F in treatment of aplastic anemia (response rates between 47% and 53%)[**41**,**42**] may be due to its different antigen source (Jurkat cells).[**42**]

## GvHD prophylaxis in allogenic HSCT

Both acute (a) and chronic (c) graft versus host disease (GvHD) are a major cause of transplant related morbidity and mortality after allogenic hematopoietic stem cell transplantation (HSCT). ATG have been used to reduce the risks of graft failure and GvHD.[**43**,**44**] Thymoglobulin is indicated for prophylaxis of acute and chronic graft versus host disease (GvHD), after hematopoietic stem cell transplantation; dosage is 2,5 mg/kg/day from day -4 to day -2 or -1.[**9**]

Several trials were performed in myeloablative conditioning regimens. A matched cohort study in patients receiving HSCT from matched unrelated donors (MUD) showed a benefit of Thymoglobulin conditioning (n=52) vs. no ATG (n=104) in transplant related mortality (TRM) (19% vs. 35%; hazard ratio (HR)=0,30; p=0,005) and overall survival (HR=0,51; p=0,03). The risk of relapse was similar in both groups.[**43**] Thymoglobulin conditioning reduced cGvHD vs. no ATG (37% vs. 60%; p=0.05) and extensive cGvHD (15% vs. 41%; p=0.01) at 5 years follow up of a randomized trial, resulting in less chronic lung dysfunction (19% vs. 51%; p=0,005) and improved quality of life, measured as Karnofsky scores ≥ 90% at 4 years (89% vs. 57%; p=0,03).[**44**] A retrospective analysis compared Thymoglobulin (n=49), ATG Fresenius (n=38) and no ATG (n=68). ATG had a positive effect on cGvHD (36% vs. 76%; p<0,0001) vs. no ATG. The better leukemia-free survival (38% vs. 21%; p=0,003) and low rates of relapse with Thymoglobulin (15% vs. 41%; p=0,014) outweighs the higher incidence of cGvHD in the Thymoglobulin group compared with ATG Fresenius.[**45**]

A single center comparison of patients undergoing allogenic HSCT from either HLA identical sibling (n=121) without or matched unrelated donor (MUD) (n=61) with Thymoglobulin conditioning showed similar survival (60% in both groups) and relapse (26,4% vs. 23%) at five years.[**46**] Low dose Thymoglobulin conditioning in matched related donors (MRD) vs. no ATG (n=54 matched pairs) resulted in lower non-relapse mortality (9% vs. 34%; p=0,002) and better overall survival (66% vs. 50%; p=0,046) despite the increased relapse rate (43% vs. 22%; p=0,05) after 4 years. PTLD and infections related to Thymoglobulin were not observed.[**47**]

Reduced intensity conditioning (RIC) protocols aim to achieve both durable donor stem cell engraftment and reduced transplant related mortality (TRM) allowing allogenic HSCT in elderly and comorbid patients. A regimen of total lymphoid irradiation plus Thymoglobulin was shown to decrease acute GvHD and to allow graft anti-tumor activity in elderly patients (n=37).[**48**] In a large retrospective analysis (n=1108, from 1994 to 2004) a regimen of fludarabine-busulfan (Flu Bu) Thymoglobulin (2x2,5 mg/kg/day) showed the best long term outcome compared to Flu Bu regimens with different ATG doses or Flu-total body irradiation (TBI).[**49**]
